# Effectiveness of the Botulinum Toxin for Treating Sialorrhea in Patients with Parkinson’s Disease: A Systematic Review

**DOI:** 10.3390/jcm8030317

**Published:** 2019-03-06

**Authors:** Juan Antonio Ruiz-Roca, Eduardo Pons-Fuster, Pia Lopez-Jornet

**Affiliations:** 1 Department of Stomatology, Faculty of Dentistry, University of Murcia, 30008 Murcia, Spain; jaruizroca@um.es; 2 Research Investigations, Instituto Murciano de Investigación Biosanitaria (IMIB), 30120 Murcia, Spain; eduardo.p.f@um.es; 3 Oral Medicine in the Department of Stomatology, Faculty of Dentistry, University of Murcia, 30008 Murcia, Spain

**Keywords:** botulinum toxin, sialorrhea, drooling, Parkinson’s disease

## Abstract

The main objective was to assess the efficacy of botulinum toxin-based treatment for sialorrhea in adult patients with Parkinson’s disease. The search was performed by using the Medline-PubMed, EMBASE and Cochrane Library databases from January 2000–December 2017, in English/Spanish in patients with Parkinson’s disease and sialorrhea. The methodological quality of trials was carried out by following the PRISMA (Preferred Reporting Items for Systematic Reviews and Meta-Analyses) criteria and the Newcastle–Ottawa Scale (NOS). Finally, a total of 21 articles were identified as fulfilling the inclusion criteria. There is no consensus regarding the site of injection of the toxin (single or multiple points), toxin dose or follow-up period. In all cases there was a reduction of sialorrhea. Treatment safety increases with the use of ultrasonography. Effects approximately occur at one week post-injection and for 3–5 months. Botulinum toxin is an effective therapeutic strategy or option in treating sialorrhea in adult patients with Parkinson’s disease. More studies with a better design, larger samples and a longer follow-up period are required to confirm these data.

## 1. Introduction

Parkinson’s disease is a chronic and progressive neurodegenerative condition with such an incidence [1] that, as the condition progresses, patients experience a deterioration of motor and non-motor symptoms [2]. The prevalence of sialorrhea in PD patients ranges from 10% to 86% [3,4,5,6,7].

Sialorrhea can lead to social and functional deficiencies, including shame and social isolation, oral and skin fungal infections, deglutition pneumonia, skin maceration, halitosis, and dehydration [2,8]. The treatment of sialorrhea in these patients aims at reducing drooling with a good risk-benefit ratio [9,10,11,12]. Treatments such as myofascial therapy and anticholinergic drugs—which inhibit the production of saliva—have a wide variety of side effects. Cheng et al. [10] find that dihydroergotoxine mesylate (α-adrenergic blocking agents) may attenuate sialorrhea in PD, showing minor adverse effects. In addition, invasive treatments such as surgery or radiotherapy are currently being offered for controlling sialorrhea [4,5,8,9].

An alternative or adjuvant treatment would be botulinum toxin, BoNT, which comprises proteases (seven serotypes, from A–G), and is produced by *Clostridium botulinum* bacteria. Such bacteria are capable of stopping the release of acetylcholine from the presynaptic nerve terminals of the neuromuscular junction, of blocking muscle contraction and the activity of the glands in the postganglionic synapses—orthosympathetic, parasympathetic. Botulinum toxin has become an important therapeutic option in the field of movement disorders, especially for focal and generalised dystonia. BoNT type A (BoNT-A) and type B (BoNT-B) are commonly used for therapeutically managing dystonia and spasticity [2,3,4,5,6,7,8,9,10,11].

Botulinum toxin injected into the parotid gland was proposed by Bushar in 1997 for treating sialorrhea. Several studies [3,6,7,12,13,14] showed strong evidence that serotypes A and B are very effective, with few side effects in various diseases, primarily in ALS (Amyotrophic Lateral Sclerosis) and PD (Parkinson’s disease).

Botulinum toxin blocks the presynaptic release of acetylcholine in parasympathetic ganglia. The effect is temporary and lasts until the presynaptic terminals regenerate, usually up to three months [15]. Accordingly, repeated injections are required [16]. Despite the importance of the application of botulinum toxin to treat sialorrhea in Parkinson’s disease, there is lack of clear consensus concerning the safest and most effective treatment method and specific clinical guidelines or recommendations.

The objective of this paper is to conduct a systematic review by analysing published clinical studies regarding the efficacy of the treatment of sialorrhea with botulinum toxin injections in patients with Parkinson’s disease.

## 2. Materials and Methods

The literature search strategy, the methods for this systematic review, as well as the study design, were determined by the authors with a protocol prior to the review process.

### 2.1. Focused Methodology 

The search strategy was performed with populations, exposures and outcomes (PEO) in order to synthesise the following question: Is the botulinum toxin therapy effective for treating sialorrhea in patients with Parkinson’s disease (Table 1)?

### 2.2. Search Strategy

Two reviewers (JARR and PLJ) independently conducted an exhaustive search of four databases: Medline-PubMed, EMBASE and Cochrane Library, following PRISMA (Preferred Reporting Items for Systematic Reviews and Meta-analyses) 2009 statements (http://www.prismastatement.org) throughout the selection process. They used a combination of keywords (“Botulinum toxin and Sialorrhea” and “Botulinum toxin and Drooling”) in “Parkinson’s disease”. Articles were limited to those published between January 2010 and December 2017, and whose studies were performed only in human beings. In the first round only titles and abstracts of retrieved articles were analysed. Then in a second round all considered eligible studies were fully examined and final decisions about inclusions were made. In case of disagreement a third reviewer (EPF) participated in order to reach consensus.

### 2.3. Selection Criteria

Clinical trials in patients over 18 years of age diagnosed with PD and sialorrhea who had been treated with intraglandular botulinum toxin to treat sialorrhea were included [1]. If the sample of the study included several neurological conditions, only PD patients were included in this study. Studies were excluded when (1) sample less than eight patients, (2) those that were published in a language different from English or Spanish, (3) meta-analysis, literature reviews, posters or communications and letters to the editor, and (4) application of botulinum toxin for indications other than sialorrhea. Efficacy, safety and doses used were collected.

### 2.4. Outcome Measurement Type

Studies assessing the effectiveness of the therapy were included in our review. They assessed such efficacy by using quality of life questionnaires, Clinical Global Impression scale, Drooling Frequency and Severity Scale, unstimulated salivary flow rate and VAS (Visual Analogic Scale).

The information extracted from each of the papers included associated pathology, sample size, study design, intervention, outcome measures, duration of follow-up process, outcomes, and side effects. Papers were classified in accordance with their level of scientific evidence by following the Newcastle–Ottawa Scale (NOS) criteria (score ranges between zero up to nine points, representing the lowest and the highest quality)

## 3. Outcomes

**Figure 1:**
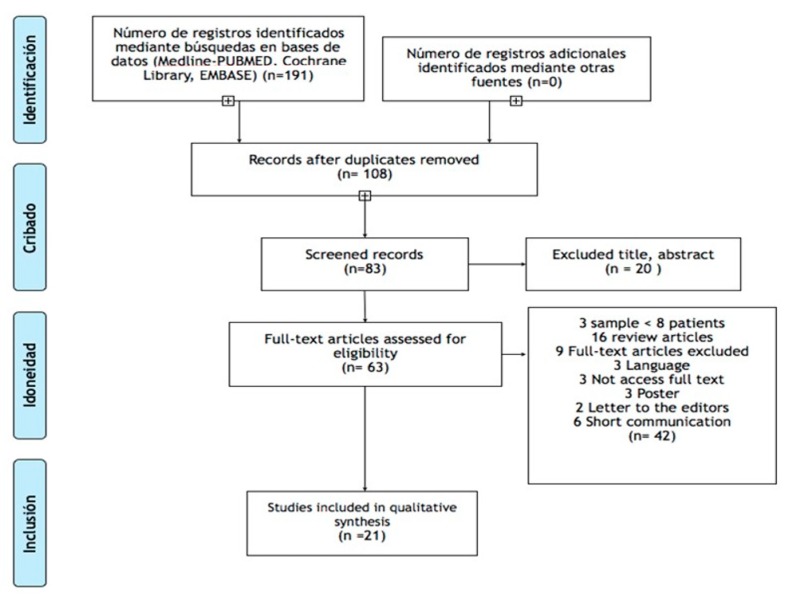
Flow diagram.

Of the 191 studies initially obtained after the keyword search, 170 were rejected for not fulfilling the inclusion criteria, so the full text of 21 documents was finally analysed (Figure 1). A fundamental inclusion criterion was that patients with sialorrhea were diagnosed with Parkinson’s disease, eight of the 21 studies analysed samples that included patients with PD and other neurodegenerative conditions, such as ALS, cerebral palsy, or encephalitis. PD comprised between 20% and 73% of the total sample. With regard to the sample size, this was variable. In only seven studies more than 30 patients with PD participated, and in one of these studies, the largest, 117 out of 160 participants suffered from this disease [2].

The total number of participants was 641. The age of the patients under study was over 53, with a mean between 68 and 70 years approximately.

Among all the salivary glands, the parotid gland is the major target when injecting botulinum toxin in all studies, and can be treated alone [6,7,16] or, what happens in most studies [3,10,14,15,17,18], along with the submandibular glands, since both produce 90% of total saliva. There are two studies [4,19] that do not specify into which gland the treatment is applied. Nevertheless, there is no consensus regarding the site of injection in the parotid gland, since some authors inject botulinum toxin in a single point, whereas others distribute the same dose in two or three different points into the same gland.

With regard to the technique for guiding the injection needle, the submandibular case is performed through palpation, and to locate the injection point in the parotid gland most studies are guided by ultrasound or ultrasonography at a frequency between 7.5 and 12 MHz, Although other authors advocate palpation and externally marking on the skin, a series of anatomical landmarks as guidance or reference points. In one study the method of guiding the injection needle is not specified [18] and in another one electromyography is utilised [2] (Table 2).

In therapeutics, two serotypes of botulinum toxin are commonly used: A (BoNT-A) and B (BoNT-B), although in the majority of studies the effectiveness of A is tested. On the other hand, there are major differences between A and B regarding the administered doses, accepting in most studies a 1:10 ratio, i.e., the equivalence of 10 U of A with 100 of B, so that a comparison between both of them can be established [30]. The dosage protocol (number of sessions, etc.) was different for each case, since no standardisation of criteria was found in the protocols.

Some authors, including Breheret et al. [17], studied the efficacy of BoNT-A, used seven different treatments combining various doses for both the parotid and submandibular glands, indicating that two ultrasound-guided injections of 15 mU in each parotid gland and one ultrasound-guided injection of 20 mU in each submandibular gland is the most effective dose and regimen.

However, Narayanaswami et al. [18] injected doses of 20 U and 30 U in the parotid and submandibular glands respectively, although no statistically significant improvement was detected.

In one study 25–40 units of BoNT-A were used in the parotid gland and 15–30 in the submandibular gland, with 40% reduction of drooling after two weeks of treatment and effectiveness within the following two months. Some years later, in 2014, these same authors conducted a similar study but with 750–2500 units of BoNT-B injected in both glands as well. A reduction of drooling between 30%–70% was observed after 6 weeks of treatment and 20% at week 12.

In another publication [13] 3 groups were created and doses were injected in the parotid and submandibular glands, increasing the doses by 50, 100 and 200 units in each group. There was greater effectiveness in the latter group, with a reduction of drooling until 24 weeks.

Two other authors [14,19], compared the effectiveness of BoNT-A with respect to BoNT-B by dosing 250 units and 2500 units respectively, in both glands—i.e., the same amount because they assumed as a conversion factor the ratio 1:10. Both therapies were equally effective and safe for the control of sialorrhea, so given a choice, these Italian authors opted for B whose costs or RRP (recommended retail price) was much lower, at least in Italy. According to these authors, the toxin additionally obtains better results in this type of underlying condition (PD) than in others, such as ALS. In these studies, neither the anticholinergic treatment nor the medication for other pathologies were discontinued or suspended, and, in general terms, it was not specified whether they had tried other treatments for sialorrhea or whether they were using one at that time and they applied the toxin as an adjuvant, so, perhaps, the results on the effectiveness of the toxin could be masked or biased.

The method to diagnose sialorrhea is very diverse and includes the measurement of the amount of excreted saliva, subjective scales or validated questionnaires like the Non-motor Symptoms Questionnaire for PD [31], the Non-motor Symptoms Assessment Scale for PD [32], the Rabboud Oral Motor Inventory for PD [33], the Drooling Rating Scale [34], the Scales for Outcomes in PD-Autonomic [35], the Sialorrhea Clinical Scale for PD [36] or the Drooling Frequency and Severity Scale [37]. In the case of DFSS—which is the most commonly used technique for studying drooling associated with other pathologies—it was considered an effective treatment when drooling was reduced by two points on the scale [20,22,23,24,25].

In relation to the adverse effects, it should be noted that in three studies no side effects were highlighted, in two of them nothing was specified in that regard, and in the rest of cases in which an alteration occurred, this was of a mild nature, for example viscous saliva, dry mouth, difficulty in the motor control of tongue, skin reaction and aggravation of previous dysphagia. [4,7,13,14,15,16,26,27,28,29].

## 4. Discussion

Sialorrhea is a severe complication in PD patients—especially in the advanced stages—so the treatment is, therefore, considered of special importance. However, throughout the review no clear and widely supported protocol was found for the effective treatment of sialorrhea in PD patients, although it seems that the best dose of BoNT-A would range between 100 U [13,14] and 250 U [11,19], and equivalent doses of BoNT-B around 2500 U [14,19] would have the same effectiveness, leaving an interval of approximately two weeks to three months between injections and using ultrasound as a guidance method, since it is safe and relatively easy to perform.

In addition, when directly comparing the doses administered in prior studies some factors, such as differences in the number of injected salivary glands and the proportion of BoNT-A injected into them influenced. Possible adverse events potentially considered and associated with BoNT-B are dry mouth, changes in saliva thickness, mild temporary dysphagia, mild weakness of mastication and diarrhoea. 

The exact injection site can be located in several manners: guided by ultrasound, electromyography or palpating the gland by previously locating a series of facial anatomical landmarks. In recent publications, like the article by Breheret et al. [17], the manner to locate this site has caused controversy. The need for ultrasound is still unclear, suggesting that in the majority of cases it is possible to inject the botulinum toxin by solely palpating the parotid gland, although it is true that ultrasonography does not involve risks or adverse events.

Many studies [3,4,7] have shown that with repeated injections, the time of effectiveness is prolonged, i.e., the mean duration of effectiveness increases along with the number of injections, and can even reach up to three years of effectiveness. One study proposes intervals of three months between injections, another one intervals of four months, whereas in another study the botulinum toxin is injected every two weeks. The rest acts according to the response to treatment. With regard to the duration of the effects of the treatment, it ranges from six weeks to seven months depending on the study reviewed and the follow-up period.

## 5. Limitations

In this systematic review an assessment of the botulinum toxin in patients with Parkinson’s disease was applied. The review resulted in the exclusion of studies which, perhaps, could have presented relevant data or outcomes regarding the use of botulinum toxin for other neurological conditions. The majority of studies additionally injected this toxin without long-term follow-up.

## 6. Conclusions

One of the major defects in the assessment of the effectiveness of the botulinum toxin-based treatment is the heterogeneity of data, since the criteria or diagnostic methodology for sialorrhea are not the same, so it is not possible to homogenise the resulting sample (type of toxin, parotid or submandibular gland) in terms of recovery or severity—most studies do not usually specify the interval between doses. In general, botulinum toxin is observed to be an effective option to treat sialorrhea in patients with Parkinson’s disease, although studies with better designs, larger samples, dose uniformity, frequency and longer follow-up period are undoubtedly necessary to offer more significant outcomes.

## Figures and Tables

**Table 1:** Population, exposure and outcomes (PEO).

Focused Question	Is the Botulinum Toxin Therapy Effective for Treating Sialorrhea in Patients with Parkinson’s Disease?
Population	Patients with Parkinson’s disease and sialorrhea
Exposure	Patient under treatment with botulinum toxin
Outcomes	Administered doses, treatment outcomes, side effects, efficacy of treatment over time

**Table 2:** Characteristics of Included Studies.

Author	Number of Patients	Study Design	Outcome Measurement	Findings	Method to Locate Glands	Side Effects
Lagalla et al. [20]	36	A double-blind, randomised, placebo-controlled study	Drooling Severity and Frequency Scale, DSFS, visuo-analogic ratings of familial distress, VAS-FD, and social distress, VAS-SD) and (saliva production by weighing dental rolls. The Global Impression Score (GIS)	BTXB represents a safe and efficacious tool for the management of PD related drooling, ensuring a long-lasting waning of this disabling symptom.	External anatomical landmarks	x
Tiigimäe-Saa et al. [21]	20	open clinical trial	Drooling was evaluated using subjective scales and objective assessment of salivary flow rate and oral health.	BNT-A injections according to the current protocol can effectively manage sialorrhea while maintaining oral health.	Ultrasound guidance	x
Svetelet al. [22].	13	open clinical trial	Activities of Daily Living of the Unified Parkinson’s Disease Rating Scale (UPDRS).	Botulinum toxin-A injections to easily accessible parotid glands, without necessity for ultrasound guidance.	Ultrasound guidance and External anatomical landmarks	x
Lagalla et al. [23]	32	double-blind, randomized placebo-controlled study	Subjective measures included a visual analogue rating of drooling frequency (VAS-D), as well as assessment of patient embarrassment within the familial (VAS-FD) and social (VAS-SD) context.	Subjects treated with BoNTX experienced a reduction in both drooling frequency and familial and social disability (Time × Group effect: *p* < 0.01), as well as in saliva production (Time × Group effect: *p* < 0.0001).	External anatomical landmarks	x
Nóbrega et al. [24]	21	open clinical trial	Drooling severity and frequency	The severity of drooling decreased in 18 (86%) patients, while frequency was reduced in 8 (38%). In 11 (52%) patients, the frequency of drooling remained constant.	Ultrasound	bilateral local oedema
Ondo, W.G. [25]	16	A double-blind placebo/treatment	The Unified Parkinson’s Disease Rating Scale (UPDRS), questionnaires regarding drooling, Visual Analogue Scale, global impressions, salivary gland imaging, and a dysphagia questionnaire.	Injections of botulinum toxin B into the parotid and submandibular glands appear to effectively improve sialorrhea without compromising dysphagia in patients with PD.	Anatomic landmarks	x
Mancini, F. [26]	20	Double-blind, placebo-controlled study	Drooling Severity and Drooling Frequency scales	BTX injection into parotids and submandibular glands, is an effective and safe treatment for drooling.	Ultrasound	x
Friedman et al. [27]	11	Cases/control	The Unified Parkinson’s Disease Rating Scale (UPDRS), saliva production by weighing dental rolls.	Botulinum toxin may be an effective and safe treatment of parkinsonian sialorrhea.	Anatomic landmarks	No
Pal et al. [28]	9	open clinical trial	Rating scales for severity and frequency of drooling and saliva production by weighing dental rolls.	Intraparotid BTX-A can be a useful, safe, and simple treatment for reducing the accumulation of saliva in neurologically impaired patients.	Anatomic landmarks	No
Santamato et al. [29]	18	open clinical trial	Questionnaire-Based Scoring System for Drool—the dose of Botox used per parotid glanding Severity and Frequency.	The severity and the frequency of drooling decreased in all patients after BTX-A after a 30-day follow-up.	Ultrasound	adverse effects could be dysphagia and chewing difficulties
Breheret et al. [17]	14	Retrospective review between May 2002 and February 2008	Quality of life questionnaire from 6–8 weeks after the injections. (0 = no efficacy, 1 = partial efficacy, 2 = good efficacy but of brief duration (<1 month), 3 = very effective: resolution of drooling, 4 = patient died or lost to follow-up).	Beneficial effect in 66% of cases. The most effective protocol was injection of 20 U of toxin into each submandibular (submaxillary) gland and 30 U into each parotid gland.	Ultrasound	No major complications were observed (haematoma of the floor of the mouth or paralysis).
Bruno et al. [4]	160	Retrospective review of patients who received treatment with injections between 1995 and 2014	Response to treatment was assessed by using a subjective Clinical Global Impression (CGI) consisting of five points (3 = very much improved, 2 = much improved, 1 = minimally improved, 0 = no change, and −1 = worse). The CGI assessment was performed after the first set of injections and in the last recorded visit.	Improvement in pain in 81% of cases, which was maintained in the last recorded visit, without significant differences with the result after the first injection. BTX treatment could play a safe and useful role in the treatment of pain in this population.	Electromyography	x
Chinnapongse et al. [6]	54	Prospective, multicentre, randomised, double-blind design	Follow-up of subjects for four weeks and up to 20 weeks. Primary measure: safety/tolerability as assessed by adverse events. Secondary measure: Efficacy, assessed by means of the Drooling Frequency and Severity Scale and unstimulated salivary flow rate.	Gastrointestinal adverse events in 31% of the active group compared to 7% in the placebo group, with dry mouth being the most common. No serious adverse events or treatment discontinuations due to adverse events. Significant improvement in DFSS at 4 weeks postinjection and decreased unstimulated salivary flow rate.	External anatomical landmarks	x
Gómez-Caravaca et al. [7]	53	Retrospective study with a long-term follow-up, between 2007 and 2013.	Variables: previous treatment, number of visits, average dose administered, duration of treatment effect and latency, assessment of response to treatment (less drooling), increased doses, mean years of follow-up and adverse events. The response to treatment was evaluated on a scale of 1 (minimal response) to 10 (maximum response), and patients were considered as responders if they scored greater than zero in this scale.	There was an improvement after treatment in 65.22% of patients with an average score of 6.85 ± 1.58 points on a scale from 0 to 10. The duration of the effect of treatment was 4.38 ± 2.11 months, with a latency period of 10.06 ± 9.63 days.	External anatomical landmarks	Mild and infrequent: skin reaction and weakness in the muscles of mastication and one case of previous dysphagia was slightly exacerbated.
Mazlan et al. [11]	30	Prospective, double-blind randomised controlled trial between September 2010 and February 2014	The primary outcome was the amount of saliva reduction, measured by the differential weight (wet *versus* dry) of intraoral dental gauze at baseline and at 2, 6, 12 and 24 weeks after injection. The secondary outcome was the subjective report of drooling by using the Drooling Frequency and Severity Scale (DFSS).	Saliva reduction in response to all doses of botulinum toxin A. No significant differences between doses. Greater mean reduction in those groups receiving the highest doses. The group that received 200 U of Dysport ® showed the greatest saliva reduction until 24 weeks and reported the most significant improvement in the DFSS score.	Ultrasound	Viscous saliva
Guidubaldi et al. [14]	14	Prospective, randomised, double-blind, crossover pilot study	Objective evaluations (cotton roll weight) and subjective evaluations (ad hoc clinical scales) were performed at baseline, after one and four weeks, and every four weeks until drooling returned to baseline.	Subjective and objective improvements in all patients. Latency was shorter upon use of botulinum toxin B (three days) compared to botulinum toxin A (six days). The mean duration of benefits was similar for bot. toxin A (75 days) and B (90 days). Either 250 U Dysport or 2500 U Neurobloc have similar effectiveness and safety in controlling sialorrhoea.	Ultrasound	Change to saliva thickness
Møller et al. [3]	12	Open, prospective study	Patients were followed up for two months with evaluations every second week by means of self-assessed rating scales for drooling intensity, discomfort and treatment effect, and determination of unstimulated whole saliva flow rate.	Drooling and saliva flow were reduced (*p* < 0.05) two weeks after treatment, without side effects. The maximal reductions during the observation period were 40% for drooling and 30% for saliva flow. There was a variation in flow. Amylase activity and total protein concentration generally increased with decreasing flow (*p* ≤ 0.03).	Ultrasound	Seven patients dropped out shortly after the first treatment due to marked worsening of their disease-related condition.
Møller et al. [15]	17		Discomfort caused by drooling was rated on a VAS (0–100), and on a scale describing frequency and severity of drooling (0 = no drooling/dry to 7 = constant drooling). Measures were obtained after 6, 12 and 18 weeks. Perception of treatment effectiveness was also measured. Saliva flow was measured with cotton rolls in 2-min collection periods. The composition of saliva was also analysed.	Number of treatment series in each patient was 1–7. Saliva flow rate and drooling were reduced 30%–70% six weeks after treatment in the first series, while sodium, chloride, and total protein increased 20–80% (*t*-tests; *p* < 0.05). After 12 weeks, drooling was still significantly reduced (20%), saliva flow tended to be, and saliva composition was back to baseline.	Ultrasound	Viscous saliva and dry mouth
Narayanaswami et al. [18]	10	Randomised, double blind, placebo-controlled crossover trial.	1. Subjects returned monthly for three evaluations after each injection. Outcome measures were saliva weight and Drooling Frequency and Severity Scale. 2. Systematic review of literature, followed by inverse variance meta-analyses using random effects models.	1. There was no significant change in the primary outcome of saliva weight one month after injection in the treatment period compared to placebo period. 2. Secondary outcomes did not change either. Meta-analysis of six studies demonstrated significant benefit of Botulinum toxin on functional outcomes. This study did not demonstrate efficacy of incobotulinum toxin A for drooling in PD, but lacked precision to exclude moderate benefit. Studies evaluating higher doses of incobotulinum toxin A into the parotid glands may be useful.	Unspecified	Difficulty during mastication and in the motor control of tongue. Viscous saliva.
Petracca et al. [19]	65	Retrospective trial	Drooling Frequency and Severity Scale four weeks after intervention	250 U of botulinum toxin A and 2500 U of botulinum toxin B are safe and effective in the treatment of sialorrhoea, even in long-term follow-up. The older the age, the longer the benefit duration. Patients with PD showed a more favourable safety-efficacy ratio than patients with ALS did.	Ultrasound	Change of saliva thickness
Sen et al. [16]	16	Retrospective analysis between February 2009 and September 2013	Severity of sialorrhoea prior to treatment was measured in accordance with Drooling Frequency and Severity Scale (DFSS). Efficacy was assessed 4 weeks after injections of BoNT-A by using DFSS and according to the subjective evaluation of patients and/or carers (caregivers).	Efficacy was 100% and mean improvement in sialorrhoea was 71.78 ± 12.95%. There was a significant difference between the first and last application regarding the mean duration of efficacy (17.28 ± 9.21 weeks and 18.03 ± 9.02 weeks, respectively, *p* = 0.001). Repeated BoNT-A injections are safe and effective in the treatment of sialorrhoea in patients with PD.	External anatomical landmarks	Not seen
